# A mathematical model of corneal endothelium pump function

**DOI:** 10.1098/rsif.2025.0167

**Published:** 2025-08-20

**Authors:** Federica Vanone, Alexander J. E. Foss, Francesco Viola, Rodolfo Repetto, Mariia Dvoriashyna

**Affiliations:** ^1^ Gran Sasso Science Institute, L'Aquila, Italy; ^2^ Department of Ophthalmology, Nottingham University Hospitals NHS Trust, Nottingham, UK; ^3^ Department of Civil, Chemical and Environmental Engineering, University of Genoa, Genoa, Italy; ^4^ School of Mathematics and Maxwell Institute, University of Edinburgh, Edinburgh, UK

**Keywords:** corneal endothelium, modelling, simulation, local osmosis, electro-osmosis, ion transport, pump and leak, endothelial water transport

## Abstract

The corneal endothelium plays a critical role in maintaining the transparency of the cornea by regulating water transport through the ‘pump and leak’ mechanism. This study presents a mathematical model to analyse fluid and ion pumping across the endothelium, accounting for two proposed mechanisms of the endothelial pump: local osmosis and electro-osmosis. The model incorporates four key ions (Na${\;}^{+}$, K${\;}^{+}$, Cl${\;}^{-}$ and HCO${,}_{3}^{-}$) and considers transcellular and paracellular transport pathways. The model predicts a water flux from the stroma to the anterior chamber as observed in experiments with isolated endothelium. Electro-osmosis is found to contribute minimally to water transport compared with local osmosis, which is the dominant mechanism. The magnitude of water flux depends on the cell membrane and tight junction permeability to water. Global sensitivity analysis reveals that water flux is also highly influenced by the tight junction permeability to different ion species, and to a smaller extent, to the permeability of cell membrane to some ions, with the specific effect depending on the ion species. The model captures experimental observations, including responses to ion channel inhibitors. This work provides a framework for understanding the factors governing fluid regulation in the cornea.

## 1. Introduction

**Figure 1.**
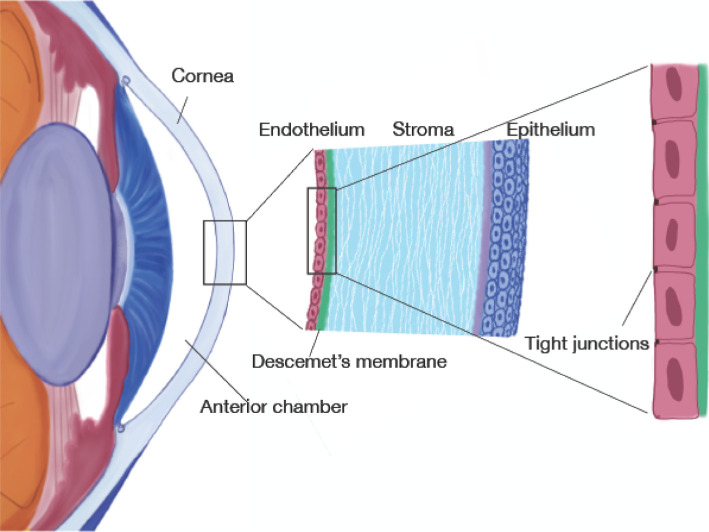
Drawing of the anterior part of the eye with zoom-in on cornea and corneal endothelium. Not to scale.

The cornea is the main refractive surface of the eye and central to its function is its transparency [1]. The cornea is located at the anterior part of the eye where it borders with the anterior chamber, see figure 1. It is composed of three main layers: the epithelium which is high resistance epithelium, constituting about 10% of the total corneal thickness; the stroma, which is the thickest layer of the cornea (about 500 μm, corresponding to about 90% of the total corneal thickness); and the endothelium, which functions as a second epithelial layer with low resistance and covers the surface facing the aqueous humour in the anterior chamber of the eye [2].

The stroma is composed of layers (*lamellae*) of collagen fibrils arranged in an extremely regular array and its transparency is due to its relatively dehydrated state, which is maintained by the corneal endothelium [3,4]. Indeed, the isolated endothelium is observed to pump water towards the anterior chamber [5]. This function of the endothelium is known as the ‘endothelial pump’, since it depends on the active transport of ions by the endothelium [6,7]. The pump has to compensate for the swelling pressure of the corneal stroma exerted by its negatively charged glycosaminoglycans. For a normal hydration of 3.4 mg per mg dry weight, this pressure has been estimated at about 60 mmHg *in vitro* [8–11], while *in vivo* measurements on rabbit corneas by Klyce *et al*. [12] show a swelling pressure of 50 mmHg. This is the pump and leak model, and the level of corneal hydration is maintained at steady state when these two processes balance [13]. Fuchs’ corneal dystrophy causes endothelial cell loss and impaired pump function, resulting in corneal swelling and loss of transparency [13]. It is one of the most common indications for a corneal graft procedure [14].

Two main mechanisms have been proposed to describe how the endothelial pump works [7]. The first is the ion secretion model, based on local osmotic effects [15], and the second is electro-osmosis [16–18]. Local osmosis was modelled in the seminal work by Diamond & Bossert [15]. Their work explains the occurrence of net water transport in absorptive or secretive epithelia as a consequence of local osmotic gradients generated by active solute transport in long, narrow, fluid-filled, membrane-bounded channels open at one end and closed at the other. In the corneal endothelium, the intercellular clefts show these characteristics. Electro-osmosis is an alternative hypothesis for explaining fluid flow in narrow channels whose surface is lined with fixed electrical charges. Such charges attract mobile ions, which accumulate in a thin electrical double layer close to the membrane. When an electric field is present along the channel, the ions will experience a Lorentz force which, since inertia is negligible, results in an equal body force on the fluid within the electrical double layer, driving the fluid flow in the channel. The cell membranes and the tight junctions are lined with negative fixed charges on their external surface, and there is a transendothelial potential of −0.5 mV [19–22] thus making the intercellular spaces and tight junctions good candidates for electro-osmosis as a fluid transport mechanism. Indeed, the experiments of Sánchez *et al.* [16] showed that there is a linear dependency between imposed transendothelial current and water flux, which further indicates the possible role of electro-osmosis.

Corneal swelling has been investigated experimentally and using mathematical models which account for the different layers of the cornea [23–27]. In these models, the mathematical treatment of the endothelial membrane is usually done by irreversible thermodynamics. Although this approach may be convenient for coupling the different layers, it does not allow for the treatment of the endothelium at the cellular level (accounting for the membrane transporters and the cleft gap), which is necessary for our purpose of investigating the relative importance of local osmosis and electro-osmosis for water transport across this cell layer.

Mathematical models of the isolated endothelium included more details about fluid and solute transport mechanisms. Liebovitch & Weinbaum [28] investigated the role of local osmotic flow by coupling transcellular (through the cell) and paracellular (along the cleft) transport. The authors considered one solute transported across the cell membranes by passive and active mechanisms and calculated the resulting osmotic water fluxes. Applied to the corneal endothelium, their model predicts net flow from the stroma to the aqueous, comparable with experimental measurement. Electro-osmosis acting within the tight junctions was modelled by Rubashkin *et al.* [17], where the authors considered the transport of NaCl and water. Fischbarg & Diecke [29] constructed a compartmental model of ion transport across the cell layer accounting for four key ions: Na${,}^{+}$ (sodium), K${,}^{+}$ (potassium), Cl${,}^{-}$ (chloride) and HCO${,}_{3}^{-}$ (bicarbonate). They included local osmosis and electro-osmosis as two experimentally observed proportionality constants relating the water flux to the ion flux and the current across the tight junction, respectively.

In this article, we develop a coupled model of fluid and solute transport across the isolated corneal endothelium, following an approach similar to that employed by some of the present authors to study water transport across the retinal pigment epithelium [30]. We relax the assumptions about the linear relationships defining local osmosis and electro-osmosis in [29]. Instead, we include a description of local osmosis similar to [28], but we also include the physical laws governing electro-osmosis in the coupling between the cell and the cleft. We consider the same four ions as in [29] and account for the presence and location of specific ion pumps, channels and transporters on the membranes of the cells, which are not part of the model by Liebovitch & Weinbaum [28]. With such a model, based on the physical principles governing water transport and accounting for the specificity of ion transport in the endothelial cells, we assess the relative importance of electro-osmosis and local osmosis in the fluid flow across the corneal endothelium. As an outlook, our model may be coupled with stromal and epithelial models, to give insights on corneal swelling accounting for the cellular structure of the endothelium.

## 2. Methods

### 2.1. Model of fluid and ion transport

#### 2.1.1. Model setup

We assume the corneal endothelium to be a continuous layer of cells separated by thin channels (clefts). This layer lies between the basal region (on the stromal side) and the apical region (towards the anterior chamber). On the apical side, the clefts are delimited by tight junctions [2,31], which we assume as selective membranes, allowing for the passage of ions and water according to specific permeabilities. In our model, we consider a two-dimensional domain that consists of one cell and half of the adjacent clefts on each side. The cell is a rectangular compartment of width w and length L (in the direction orthogonal to the endothelium plane). The cleft is a rectangle of width h and length L.

**Figure 2.**
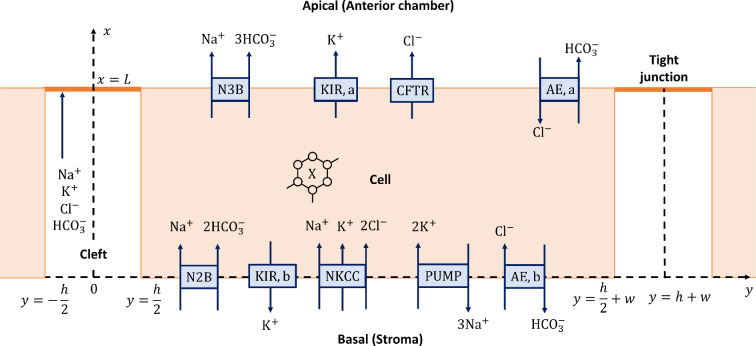
The domain is [0,L]×[0,h+w]. The ion species and the ion transporters on the membrane are shown. X indicates the fixed negative charge present in the cell. Not to scale.

We introduce a coordinate system (x,y), with the x axis going from the basal to the apical region and the y axis is orthogonal to it (figure 2). In this coordinate system, the domain is [0,L]×[0,h+w]. The cell is [0,L]×[h/2,h/2+w] and two half-clefts are [0,L]×[0,h/2] on the left of the cell and [0,L]×[h/2+w,h+w] on the right. The tight junctions are at {L}×[0,h/2] and {L}×[h/2+w,h+w]. The tight junctions divide the cell membrane into two parts: the apical membrane ({L}×[h/2,h/2+w]), separating the cell from the apical region (x≥L), and the basolateral membrane ([0,L]×{h/2})∪({0}×[h/2,h/2+w])∪([0,L]×{h/2+w}), separating the cell from the clefts and the basal region (x≤0). In our notation, each compartment will be associated with a letter as in table 1.

**Table 1.** Notation for the compartments and ions. The letters are associated with compartments and are used as superscripts, and the numbers are associated with the ion species and are used as subscripts.

(a) compartments		(b) ions	
compartment	letter	ion	number
cell	c	Na^+^	0
cleft (lateral)	l	K^+^	1
apical	a	Cl^−^	2
basal	b	${\text{HCO}}_{3}^{-}$	3

We consider all the compartments are filled with water and salt solution, which dissociates into four ions: Na${,}^{+}$ (sodium), K${,}^{+}$ (potassium), Cl${,}^{-}$ (chloride) and HCO${,}_{3}^{-}$ (bicarbonate). We choose the cations and the anions with the highest concentration in the aqueous humour [32], for which there is evidence about membrane transporters regulating their traffic among compartments, as described later in this section. We denote these ions with numbers as in table 1. The concentration of ion i∈{0,1,2,3} (with valence $z_{i}$) in the region m∈{c,l,a,b} is denoted as $c_{i}^{m}$. The cell also has a fixed negative charge X, with concentration $c_{X}$ and valence $z_{X}$. We assume the endothelium to be isolated (stroma removed) and immersed in an isotonic solution, so we prescribe the same concentrations in the apical and basal region for each ion, as specified in table 2.

**Table 2.** Parameters.

parameter	description	value	reference
universal constants			
F	Faraday constant	96 485.332 C mol^−1^	
R	universal gas constant	8.314 J mol^−1^ K^−1^	
valence			
$z_{0}$	valence of Na${,}^{+}$	+1	
$z_{1}$	valence of K${,}^{+}$	+1	
$z_{2}$	valence of Cl${,}^{-}$	−1	
$z_{3}$	valence of HCO${,}_{3}^{-}$	−1	
$z_{X}$	valence of the fixed charges in the cell	−1.05	estimated
aqueous solution			
T	standard body temperature	307 K	[33]
ϵ	aqueous dielectric permittivity	$73\times 8.854\times 10^{-12}$ C V^−1^ m^−1^	[34]
μ	dynamic viscosity of water at 34°C	$7.34\times 10^{-4}$ Pa s	
geometry			
w	cell width	20 µm	[35]
L	cell/cleft length	5 µm	[35]
h	cleft width	30 nm	[35,36]
$\lambda_{D}$	debye length	1.33 nm	computed
cell membrane and tight junction			
$\sigma_{0}$	cell membrane charge density	−0.0048 C m^−2^	estimated
$C_{m}$	cell membrane capacitance per unit area	$10^{-2}$ F m^−2^	[37]
$L_{p}$	cell membrane permeability	$2\times 10^{-12}$ m Pa^−1^ s^−1^	[38–41], [40]
$L_{TJ}$	tight junction permeability	$10L_{p}$	assumed
diffusion coefficients			
$D_{0}$	Na${,}^{+}$ diffusion coefficient	$3.00\times 10^{-9}\;{\text{m}}^{2}$ s^−1^	estimated
$D_{1}$	K${,}^{+}$ diffusion coefficient	$2.22\times 10^{-9}\;{\text{m}}^{2}$ s^−1^	estimated
$D_{2}$	Cl${,}^{-}$ diffusion coefficient	$1.69\times 10^{-9}\;{\text{m}}^{2}$ s^−1^	estimated
$D_{3}$	HCO${,}_{3}^{-}$ diffusion coefficient	$1.36\times 10^{-9}\;{\text{m}}^{2}$ s^−1^	estimated
apical and basal ion concentrations			
$c_{0}^{a}=c_{0}^{b}$	Na${,}^{+}$ apical/basal concentration	140.1 mM	[29]
$c_{1}^{a}=c_{1}^{b}$	K${,}^{+}$ apical/basal concentration	4.9 mM	[29]
$c_{2}^{a}=c_{2}^{b}$	Cl${,}^{-}$ apical/basal concentration	108 mM	[29]
$c_{3}^{a}=c_{3}^{b}$	HCO${,}_{3}^{-}$ apical/basal concentration	37 mM	[29]

The ions are transported across the cell membranes by the ion channels and transporters, as shown in figure 2. The apical and the basolateral membranes exhibit different transporters. On the basolateral membrane, we included the Na${,}^{+}$−2HCO${,}_{3}^{-}$ cotransporter (N2B) [7,29,42,43], the K${,}^{+}$ channel (KIR,b) [29,44,45], the Na${,}^{+}$–K${,}^{+}$−2Cl${,}^{-}$ cotransporter (NKCC) [7,29,46–48], the Cl${,}^{-}$–HCO${,}_{3}^{-}$ exchanger (AE,b) [7,29] and the Na${,}^{+}$–K${,}^{+}$ pump (PUMP) [7,29,49–51], which actively transports 2K${,}^{+}$ molecules into the cell and three molecules of Na${,}^{+}$ out of the cell. On the apical membrane, we included the Na${,}^{+}$–3HCO${,}_{3}^{-}$ cotransporter (N3B) [29,43], the K${,}^{+}$ channel (KIR,a) [29,45], the Cl${,}^{-}$ channel (CFTR) [7,29,52] and the Cl${,}^{-}$–HCO${,}_{3}^{-}$ exchanger (AE,a). This last one was included because there is no evidence of it being present only on the basolateral side. We did not include a functional epithelial sodium channel (ENaC) in the apical membrane considered in [29], as this channel is usually located in tight epithelia [53] and not leaky as here. Furthermore, recent expression data indicate that the corneal endothelium is missing some subunits for this channel [54], and combined with the presence of CFTR which is known to inhibit ENaC, all argue for not including it. The tight junction is permeable to all the ions in the solution with different permeability values [55]. The transport of ions will depend on the concentration and electrical potential difference across the membranes. We therefore introduce electrical potential in region m∈{c,l,a,b}, which we denote with $V^{m}$.

Water is transported across the cell membranes and the tight junctions according to Starling law [56], thus taking into account both mechanical and osmotic pressure differences. We also consider electro-osmosis in the cleft.

In what follows, we will describe separately the problem in the cell and in the cleft. The variables are listed in table 3 and include concentrations, electrical potentials, hydrostatic pressure and fluid velocity in the cleft. The cell is assumed to be well mixed and it is described as a zero-dimensional domain, and thus each variable is assumed to be constant there. On the other hand, variables are spatially variable in the cleft gap. We will refer to $v^{l}=\left(c_{0}^{l},c_{1}^{l},c_{2}^{l},c_{3}^{l},V^{l},p,u_{x},u_{y}\right)$ as the vector of cleft variables, and to $v^{c}=\left(c_{0}^{c},c_{1}^{c},c_{2}^{c},c_{3}^{c},c_{X},V^{c},V^{a}\right)$ as the vector of cell variables.

**Table 3.** m∈{c,l,a,b}, i∈{0,1,2,3}. We consider $p^{m}=0$ for m∈{c,a,b}. The gauge value for the potential is $V^{b}=0$.

variable	description
$c_{i}^{m}$	concentration of ion i in compartment m , m∈{c,l}
$c_{X}$	fixed charges concentration in the cell
$V^{m}$	electrical potential difference in region m with respect to the basal, m∈{c,l,a}
$p^{m}$	fluid pressure in region m
$u=\left(u_{x},u_{y}\right)$	fluid velocity in the cleft

#### 2.1.2. Transport in the cell

**Figure 3.**
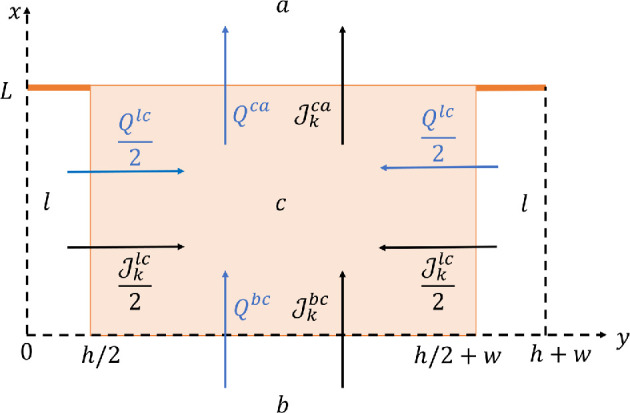
$J_{k}^{ij}$ is the flux of ion k from compartment i to j, according to the notation in table 1. The blue arrows show the positive direction of the water flow rate across the cellular membrane (${\text{m}}^{2}\;{\text{s}}^{-1}$). Note that if the solutions in a and b are the same, $Q^{bc}=-Q^{ca}$.

The problem in the cell is based on the conservation of solutes and water fluxes and electroneutrality. The flux of ion k from compartment i to j, $J_{k}^{ij}$, across a membrane or tight junction is the sum of the fluxes of that ion across each channel allowing for its passage (figure 3). The specific expressions of $J_{k}^{ij}$ are reported in electronic supplementary material, section S3. The fluxes across each channel depend on the potential and concentration differences across the membrane and on permeability coefficients ($P_{KIR,b}$, $P_{KIR,a}$, $P_{CFTR}$, $P_{PUMP}$, $P_{KNCC}$, $P_{N2B}$, $P_{AE,a}$, $P_{N2B}$, $P_{AE,a}$, $P_{i}^{TJ}$, i=0,…,3) that are treated as input parameters [57]. The expressions for all the fluxes are given in electronic supplementary material, table S3. The concentration of ions in the basal and apical region are fixed *a priori* as in [29], in order to have isotonic solutions in the two sides of the endothelium. We impose a balance of ion concentrations at steady state in the cell for all the ions, accounting for ion fluxes through the apical and the basolateral membranes:


(2.1) $$wJ_{i}^{bc}+2\int_{0}^{L}J_{i}^{lc}\text{d}x-wJ_{i}^{ca}=0,\;i=0,1,2,3.$$

Here, the fluxes across the basal and the lateral sides of the membrane are considered as separate, since they depend on concentrations and potentials, which are not the same in the two domains. $J_{i}^{lc}$ is the flux across each of the lateral membranes [0,L]×{h/2} and [0,L]×{h/2+w}. Since we consider the system to be symmetric, the contribution is the same on both sides, therefore the flux across the lateral membranes is $2\int_{0}^{L}J_{i}^{lc}\text{d}x$. $J_{i}^{lc}$ involves the cleft variables, thus it depends on x.

The fluid velocity across a membrane or tight junction from compartment i to j (in m s^−1^), $v_{osm}^{ij}$, is driven by hydrostatic and osmotic pressure difference between compartment i and j:


(2.2) $$\begin{array}{lcr} & v_{osm}^{ij}=L_{p}^{ij}(\Delta p^{ij}+\Delta \pi^{ji}), & \end{array}$$

where $L_{p}^{ij}$ is the permeability of the membrane to water, $\Delta p^{ij}=p^{i}-p^{j}$ is the mechanical pressure difference across the membrane between i and j, $\Delta \pi^{ji}=RT\sum_{k}(c_{k}^{j}-c_{k}^{i})$ is the osmotic pressure jump across the membrane between the same two compartments for i,j≠c, according to Van’t Hoff’s law [56]. R is the universal gas constant and T the absolute temperature. When one of the two compartments is the cell, we need to account also for the fixed charges in the cellular osmolarity: $\Delta \pi^{ci}=RT(c_{X}+\sum_{k}(c_{k}^{c}-c_{k}^{i}))$. We assume all sides of the cell membrane are equally permeable to water, thus $L_{p}=L_{p}^{ij}$ ∀i,j∈c,l,a,b. Since we are considering an isolated endothelium, with no pressure jump across it, $p^{a}=p^{b}=0$. We also assume $p^{c}=0$.

The overall water flow rates (per unit length) across each portion of the cell membrane (in m${,}^{2}$ s^−1^) accounting also for the membrane extension are $Q^{bc}=wv_{osm}^{bc}$, $Q^{ca}=wv_{osm}^{ca}$, $Q^{lc}=2\int_{0}^{L}v_{osm}^{lc}\text{d}x$. The water balance in the cell, accounting for the ion fluxes across the basolateral and apical membrane and for the symmetry of the clefts, is $Q^{bc}+Q^{lc}-Q^{ca}=0$, or


(2.3) $$wL_{p}RT(c_{X}+\sum_{i=0}^{3}(c_{i}^{c}-c_{i}^{b}))+2\int_{0}^{L}L_{p}(p^{l}+RT(c_{X}+\sum_{i=0}^{3}(c_{i}^{c}-c_{i}^{l})))\text{d}x-wL_{p}RT(\sum_{i=0}^{3}(c_{i}^{a}-c_{i}^{c})-c_{X})=0.$$

Near the cell membrane, which is negatively charged, there is an excess of charge that accumulates in the Debye layer, of length $\lambda_{D}\approx 1$ nm. Since the Debye layer is small compared to the characteristic cell thickness w=20 µm, we can assume electroneutrality in the intracellular medium (away from the cell membranes) [58,59]:


(2.4) $$\begin{array}{lcr} & z_{X}c_{X}+\sum_{i=0}^{3}z_{i}c_{i}^{c}=0. & \end{array}$$

We now have six equations for the six unknowns in the cell: the ion concentrations $c_{i}^{c}$ for i=0,1,2,3, the fixed charges concentration $c_{X}$ and the potential $V^{c}$.

It is of interest to consider TEP (transepithelial potential, $V^{a}$) as a variable as well. We therefore need another equation, assuming a gauge value of 0 for $V^{b}$. We thus add the open circuit condition to close the system. This means that the overall flux of ions crossing the epithelium (apical cell membrane and tight junction) should be electroneutral:


(2.5) $$\sum_{i=0}^{3}z_{i}J_{i}^{TJ}+\sum_{i=0}^{3}z_{i}J_{i}^{ca}=0.$$

Here, we denote $J_{i}^{TJ}=\frac{h}{w}J_{i}^{la}$. This condition is often used in experiments on the isolated endothelium [16].

Overall, the resulting system consists of seven nonlinear algebraic equations in the seven cellular variables ($c_{i}^{c}$, i=0,1,2,3, $V^{a}$, $V^{c}$, $c_{X}$). The cleft variables ($c_{i}^{l}$, i=0,1,2,3, $V^{l}$, $p^{l}$, $u_{x}$, $u_{y}$) appearing in the equations are additional unknowns. As described in §2.1.5, when coupling the transport problems in the cell and in the cleft, the value of $v^{l}$ will be obtained as the solution of the problem in the cleft, thus we consider $v^{l}$ to be known when solving for the transport in the cell.

All concentrations are assumed to be the same in the apical and basal regions and their values are taken to be known (table 2).

#### 2.1.3. Transport in the cleft

The flux of ion i in the cleft includes electrodiffusion and advection [57]:


(2.6) $$\begin{array}{lcr} & j_{i}=\left[\begin{array}{c} j_{ix}\\ j_{iy} \end{array}\right]=-D_{i}(\nabla c_{i}^{l}+z_{i}\frac{F}{RT}c_{i}^{l}(\nabla V^{l}))+uc_{i}^{l}, & \end{array}$$

where $D_{i}$ is the diffusion coefficient of ion i, F the Faraday constant, ∇=(∂/∂x,∂/∂y) and $u=\left(u_{x},u_{y}\right)$ is the fluid velocity. Since the considered species do not interact chemically, conservation of mass in the cleft for each ion i at steady state reads


(2.7) $$\begin{array}{lcr} & \nabla \cdot j_{i}=0,\;\forall i=0,\ldots ,3. & \end{array}$$

The electrical potential is governed by the Poisson equation


(2.8) $$\begin{array}{lcr} & \nabla^{2}V^{l}=-\frac{\rho }{\epsilon }, & \end{array}$$

where ϵ is the dielectric constant of water at 34°C and $\rho =F\sum_{i=0}^{3}z_{i}c_{i}^{l}$ is the net charge density.

The fluid velocity $u=\left(u_{x},u_{y}\right)$ and pressure $p^{l}$ in the cleft are governed by the stationary Stokes equations. To account for electro-osmosis, we include the Lorentz force on the ions $b=\rho \nabla V^{l}$ as a body force in the momentum equation [57]:


(2.9) $$\begin{array}{lcr} & -\nabla p^{l}+\mu \nabla^{2}u+\rho \nabla V^{l}=0, & \end{array}$$

with μ the dynamic viscosity of water. The last equation of the cleft system is the continuity equation


(2.10) $$\begin{array}{lcr} & \nabla \cdot u=0. & \end{array}$$

**Figure 4.**
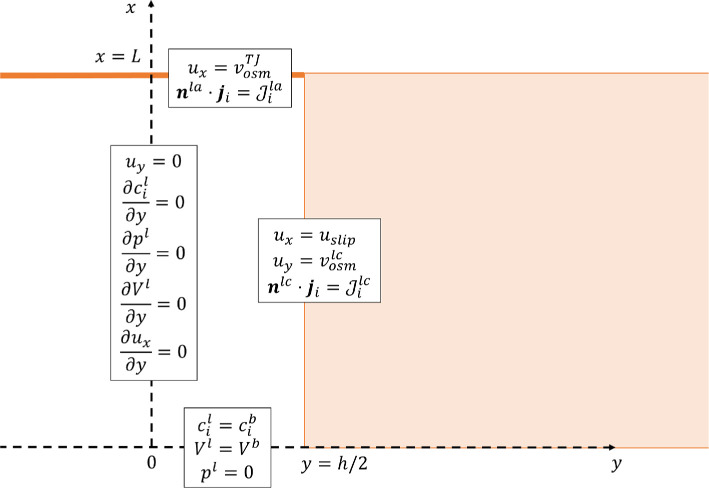
The figure shows the boundary conditions at y=0 (equations (2.16) and (2.17)), at y=h/2 (equations (2.18), (2.19) and (2.20)), at x=0 (equations (2.11), (2.12) and (2.13)), and at x=L (equations (2.14) and (2.15)).


Since the two clefts are symmetric, we study only the left half [0,L]×[0,h/2]. As boundary conditions (figure 4), at the inlet of the cleft (x=0) we prescribe that ion concentrations, potential and pressure match the corresponding values in the basal region:


(2.11) $$\begin{array}{lcr} & c_{i}^{l}(x,y)=c_{i}^{b},\;i=0,\ldots ,3\;(x=0); & \end{array}$$

(2.12) $$\begin{array}{lcr} & V^{l}(x,y)=V^{b}\;(x=0); & \end{array}$$

(2.13) $$\begin{array}{lcr} & p^{l}(x,y)=0\;(x=0). & \end{array}$$

At the tight junction (x=L), we impose the flux of each ion i is equal to $J_{i}^{la}$ as in electronic supplementary material, equation (S5):


(2.14) $$\begin{array}{lcr} & n^{la}\cdot j_{i}{|}_{x=L}=J_{i}^{la}\;i=0,1,2,3, & \end{array}$$

where $n^{la}=\left[\begin{array}{c} 1\\ 0 \end{array}\right]$ is a unit vector, normal to the tight junction and pointing towards the apical region.

For the fluid flux, we impose Starling law [56]:


(2.15) $$\begin{array}{lcr} & u_{x}(x,y)=v_{osm}^{TJ}:=L_{p}^{TJ}[RT\sum_{i=0}^{3}(c_{i}^{a}-c_{i}^{l}(x,y))+p^{l}(x,y)]\;(x=L). & \end{array}$$

Since the cleft is symmetric with respect to y=0, and we impose symmetry conditions in y=0:


(2.16) $$\begin{array}{lcr} & u_{y}(x,y)=0\;(y=0), & \end{array}$$

and the y-derivatives of all the cleft variables must be 0 at y=0:


(2.17) $$\begin{array}{lcr} & \frac{\partial c_{i}^{l}}{\partial y}(x,y)=0,\;\frac{\partial p^{l}}{\partial y}(x,y)=0,\;\frac{\partial V^{l}}{\partial y}(x,y)=0,\;\frac{\partial u_{x}}{\partial y}(x,y)=0,\;(y=0) & \end{array}$$

for i=0,1,2,3.

On the lateral cell membrane (y=h/2), we impose the ion fluxes


(2.18) $$\begin{array}{lcr} & n^{lc}\cdot j_{i}{|}_{y=h/2}=J_{i}^{lc},\;i=0,1,2,3, & \end{array}$$

with $n^{lc}$ a unit vector, normal to the lateral cell membrane, pointing towards the cell. For the water flux, we impose a slip condition


(2.19) $$u_{x}(x,y)=u_{slip}(x)\;(y=h/2),$$

with $u_{slip}$ being determined as a modified Helmholtz–Smoluchowski slip velocity for electro-osmotic flows (electronic supplementary material, section S6). $u_{slip}$ depends on the cleft concentrations and potential and on the cellular potential. For the y-component of the fluid velocity on the lateral membrane, we impose osmotic flux:


(2.20) $$u_{y}(x,y)=v_{osm}^{lc}(x,y)\;(y=h/2),$$

where $v_{osm}^{lc}:=L_{p}[RT(X+\sum_{i=0}^{3}(c_{i}^{c}-c_{i}^{l}(x,y)))+p^{l}(x,y)].$

The condition of the electrical potential at the lateral membrane is introduced in electronic supplementary material, section S6, based on [59]. It relates the electric field with the potential difference across the membrane and the surface charge density through the membrane capacitance and dielectric constant.

We define some quantities that will be useful in the interpretation of the results: the flow rate (in m${,}^{2}$ s^−1^) across the tight junction (from the lateral to the apical compartment)


(2.21) $$Q^{la}=\int_{0}^{h/2}u_{x}(L,y)\text{d}y+\int_{h/2+w}^{h+w}u_{x}(L,y)\text{d}y=2\int_{0}^{h/2}u_{x}(L,y)\text{d}y,$$

and at the inlet of the cleft (from the basal to the lateral compartment)


(2.22) $$Q^{bl}=\int_{0}^{h/2}u_{x}(0,y)\text{d}y+\int_{h/2+w}^{h+w}u_{x}(0,y)\text{d}y=2\int_{0}^{h/2}u_{x}(0,y)\text{d}y.$$

The transendothelial water flow rate (per unit length) will be $Q=Q^{la}+Q^{ca}$, in m${,}^{2}$ s^−1^. The corresponding flux (flow rate per unit surface) is $\bar{Q}=\frac{Q}{w+h}$, in m s^−1^.

#### 2.1.4. Model simplification in the cleft

In this section, we describe the simplification procedure to reduce the cleft equations to a system of ODEs in the variable x. The detailed derivation is reported in electronic supplementary material, section S5. Here, we use the notation $p=p^{l}$, $V=V^{l}$, $c_{i}=c_{i}^{l}$. We split the cleft in two domains: the electrical double layer (EDL) within 1 Debye length from the cell membranes, and the electroneutral bulk.

We scale the cleft equations and solve them using an asymptotic expansion in terms of η=h/L, where L and h are the length scales for x and y, respectively. At leading order in η, pressure p, concentrations $c_{i}$ and potential V are independent of y.

The equations for the conservation of ions are derived at the second order in the asymptotic expansion of (2.7) in terms of η:


(2.23) $$0=-\frac{\partial }{\partial x}(\frac{\partial c_{i}}{\partial x}+z_{i}\frac{F}{RT}\frac{\partial V}{\partial x}c_{i})+\frac{2}{D_{i}h}\frac{\partial c_{i}}{\partial x}\int_{0}^{h/2}u_{x}\text{d}y-\frac{2}{D_{i}h}c_{i}v_{osm}^{lc}+\frac{2}{D_{i}h}J_{i}^{lc},$$

for i=0,1,2,3, with $v_{osm}^{lc}$ from (2.20).

In the bulk, electroneutrality holds, meaning that ρ=0. Therefore, we drop equation (2.23) for i=3 and the Poisson equation (2.8). We use instead electroneutrality $c_{3}=c_{0}+c_{1}-c_{2}$ and the linear combination $S_{0}+S_{1}-S_{2}-S_{3}=0$, where we call $S_{i}$ equation (2.23) for ion i.

Since ρ=0, the momentum equation (2.9) becomes


(2.24) $$\begin{array}{lcr} & -\nabla p+\mu \nabla^{2}u=0. & \end{array}$$

By integrating the momentum equation (2.24) twice with respect to y, and applying the symmetry condition (2.17) at y=0 and the slip condition (2.19) at y=h/2, we obtain an expression for $u_{x}$:


$$u_{x}=\frac{1}{2\mu }\frac{\partial p}{\partial x}(y^{2}-(\frac{h}{2}{)}^{2})+u_{slip},$$

where $u_{slip}$ is the slip velocity and must be found by matching the solution in the bulk with the one in the EDL.

An equation for the pressure is obtained by integrating the continuity equation (2.10) with respect to y from y=0 to y=h/2, applying the symmetry condition at y=0 and imposing the flux across the cell membrane. The resulting equation for pressure is


(2.25) $$\frac{\partial^{2}p}{\partial x^{2}}=\frac{3\mu }{(h/2{)}^{2}}(\frac{v_{osm}^{lc}}{h/2}+\frac{\partial u_{slip}}{\partial x}).$$

For determining $u_{slip}$, we solve the equations in the EDL (details in electronic supplementary material, section S6). Here, electroneutrality does not hold, so we need to solve equations (2.7), (2.8), (2.9) and (2.10). The scaling is the same as for the bulk, but for the y variable, which in the EDL scales with the Debye length $\lambda_{D}=\sqrt{\frac{\epsilon RT}{F^{2}C}}$. We perform an asymptotic expansion in terms of $\delta =\frac{\lambda_{D}}{L}$. The matching between the bulk and EDL solution provides the expression for the slip velocity (electronic supplementary material, equation (S70)).

#### 2.1.5. Implementation

The numerical solution is computed in Matlab [60]. We use the notation introduced in §2.1.1, with a vector for the cleft variables $v^{l}=\left(c_{0}^{l},c_{1}^{l},c_{2}^{l},c_{3}^{l},V^{l},p,u_{x},u_{y}\right)$ and one for the cell variables $v^{c}=\left(c_{0}^{c},c_{1}^{c},c_{2}^{c},c_{3}^{c},c_{X},V^{c},V^{a}\right)$. The cellular equations (2.1), (2.3), (2.4), (2.5) are solved using vpasolve Matlab function by the function $v^{c}=\text{cell\_solution}(v^{l})$, whereas the cleft ODEs are solved using bvp5c (Matlab function implementing a fifth-order collocation method) by the function $v^{l}=\text{cleft\_solution}(v^{c})$. In the supplementary material, we report the system of cleft equations in the form of first-order ODEs (S78), and the corresponding boundary conditions (S79). Since the cell variables are inputs for the cleft equations and vice versa, and since we look for $v^{c}$ and $v^{l}$ simultaneously satisfying the equations in both domains, we develop an iterative procedure to couple the cell and cleft solutions, described below and illustrated in a flow chart in electronic supplementary material, section S8:

(i) We define the parameter values in table 2 and channel permeabilities in electronic supplementary material, table S4.
(ii) We initialize the cleft variables $v^{l}=v_{0}^{l}$ to their values in x=0 as in (2.11), (2.12), (2.13).
(iii) We define the cell error (err_cell) as the maximum among all the cell variables in $v^{c}$ of the $L_{1}$-norm of the relative difference between two consecutive iterations. Analogously we define the cleft error (err_cleft). We set the tolerance (toll) and a maximum number of iterations (N).
(iv) We compute the cell solution to obtain the initial values for the cell variables $v_{0}^{c}=\text{cell\_solution}(v_{0}^{l})$.
(v) We iterate until the error is lower than the tolerance:
  for i = 1:N
    $v_{i}^{l}=\text{cleft\_solution}(v_{i-1}^{c})$;
    $v_{i}^{c}=\text{cell\_solution}(v_{i}^{l})$;
    compute err_cell and err_cleft between $v_{i}^{l}$ and $v_{i-1}^{l}$ and err_cell between $v_{i}^{c}$ and $v_{i-1}^{c}$
    if err_cleft < toll and err_cell < toll
      break
    end
  end

The mesh given to bvp5c and used for integration consists of the zeros of the Chebyschev polynomials in [0,1], and the integration weights are computed by the Clenshaw–Curtis formula [61,62].

### 2.2. Parameters

Many model parameters are taken from literature or estimated (table 2). The detailed explanation of the choice of each parameter is in electronic supplementary material, section S1.

The channel permeabilities used for the reference case simulation are found by least squares optimization to match the literature values for $c_{0}^{c}$, $c_{1}^{c}$, $c_{2}^{c}$, $c_{3}^{c}$, $V^{a}$, $V^{c}$ (electronic supplementary material, table S1). The permeabilities we use for the reference case are in set 1 in electronic supplementary material, table S4, together with an alternative set (set 2). The details about the procedures and the cost functions used to obtain the two sets of parameters are described in electronic supplementary material, section S4.

### 2.3. Sensitivity analysis

To assess the contribution of the channel permeabilities to the model outputs, we perform a global sensitivity analysis using the eFAST method [63,64]. This method provides an efficient way for sampling the parameter space by a suitable search curve and for computing the total sensitivity index $S_{T_{i}}$ for each input i. These describe the effect of input i on the output, accounting also for the interactions with other inputs, in an ANOVA-like variance decomposition framework. In particular, $S_{T_{i}}$ is the sum of the sensitivity indices of all the orders involving input i (the first-order index for input i, $S_{i}$, is the fraction of the variance of the output due to input i, the second-order index $S_{ij}$ is the fraction of the variance due to the interaction between the two inputs i,j which cannot be explained by the sum of the first order terms, the nth order index $S_{i_{1}\ldots i_{n}}$ is the fraction of the variance due to the interaction among $i_{1}\ldots i_{n}$ which cannot be explained by lower-order terms).

As model outputs, we choose the cellular concentrations $c_{i}^{c}$ for i=0,…,3 and $c_{X}$, the cellular potential $V^{c}$, the transendothelial potential $V^{a}$, the ion fluxes across the tight junction $J_{i}^{TJ}$ and across the whole endothelium $J_{i}=J_{i}^{TJ}+J_{i}^{ca}$ for i=0,…,3 and the transendothelial water flux $Q=Q^{ca}+Q^{la}$ for a total of 16 outputs. The input parameters are the permeabilities $P_{KIR,b}$, $P_{KIR,a}$, $P_{CFTR}$, $P_{KNCC}$, $P_{N2B}$, $P_{N3B}$, $P_{i}^{TJ}$, i=0,…,3. We fix $P_{PUMP}$ because the Na${,}^{+}$–K${,}^{+}$ pump is the only active transporter and we want to keep its effect constant while investigating the behaviour of the other components of the system. We fix also $P_{AE,b}$ and $P_{AE,a}$, since according to a sensitivity analysis performed on the cellular domain, none of the considered outputs is sensitive to them (electronic supplementary material, section S9). As prior distribution for each parameter, we assume a uniform distribution centred in the values of set 1 electronic supplementary material, table S4. We include also a dummy parameter, which does not appear in the equations, with a uniform distribution centred in 0. Since the sensitivity of the outputs the dummy parameter should be 0, we use it as a control. Sensitivity indices lower than the sensitivity to the dummy parameter should not be considered significantly different from 0. Overall, we consider 11 inputs (10 permeabilities and the dummy parameter) and 16 outputs. We use three search curves, with 1000 sampling points each.

## 3. Results

### 3.1. Reference case

Here, we present the results of the model simulations with the input parameters of set 1 in electronic supplementary material, table S4, and with a tolerance toll= $10^{-8}$ for the numerical solver. The computed cellular concentrations are $c_{0}^{c}=14.7$ mM, $c_{1}^{c}=132.1$ mM, $c_{2}^{c}=38.2$ mM, $c_{3}^{c}=25.1$ mM. These values are close to the optimization targets reported in electronic supplementary material, table S1.

**Figure 5.**
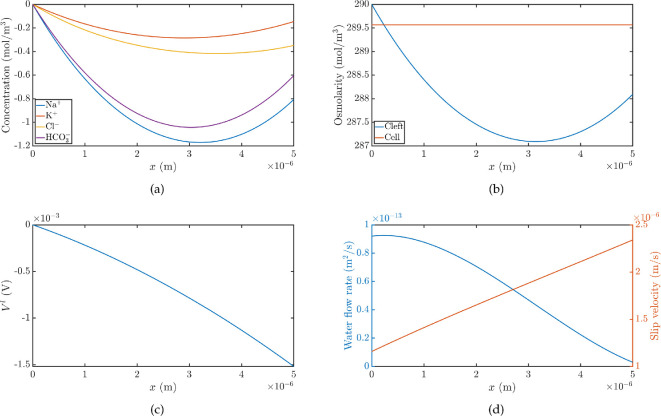
(a) Concentration difference between the cleft and the basal domain $c_{i}^{l}-c_{i}^{b}$ for the four ion species i=0,1,2,3 along the cleft. The boundary with the basal region is at x=0 and the tight junction is at x=L. (b) Osmolarity in the cleft ($\sum_{i=0}^{3}c_{i}^{l}$) and in the cell ($\sum_{i=0}^{3}c_{i}^{c}+c_{X}$). (c) Electrical potential in the cleft, $V^{l}$. (d) Left axis, water flow rate along the cleft, computed as $2\int_{0}^{h/2}u_{x}(x,y)\text{d}y$. The first point of the curve is $Q^{bl}=2\int_{0}^{h/2}u_{x}(0,y)\text{d}y$ and the last point is $Q^{la}=2\int_{0}^{h/2}u_{x}(L,y)\text{d}y$. Right axis, slip velocity ($u_{slip}$).

Figure 5a shows that the concentrations of all the species in the cleft are below their values at x=0 (inlet of the cleft). The osmolarity in the cleft starts from a value of 290 mM at x=0 as in the stroma, slightly higher than the cellular osmolarity (289.6 mM). Since the cleft concentrations decrease, the cleft osmolarity soon becomes lower than the cellular one (figure 5b). After reaching their minima along the cleft, concentrations rise again (figure 5a) due to the high permeability of the tight junctions, but overall the osmolarity along the cleft stays below the cellular value.

**Figure 6.**
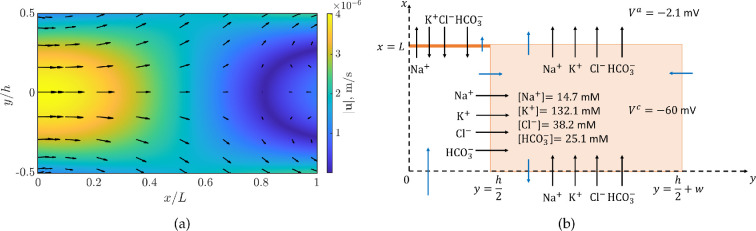
(a) Contour plot of $\sqrt{u_{x}^{2}+u_{y}^{2}}$. The vectors are u(x,y). (b) The arrows represent the direction of the ions (black arrows) and fluid flux (blue arrows) across the cellular membrane and tight junction. The definition of the fluxes is in figure 3. The magnitude of the fluxes is in table 4. The cellular concentrations of the ions and the cellular potential are reported, as well as the transendothelial potential.

The cellular potential is $V^{c}=-60$ mV, the transendothelial potential is $V^{a}=-2.1$ mV, a value larger than that reported in electronic supplementary material, table S1. In the cleft, the electrical potential becomes increasingly negative from x=0 to x=L, with a potential jump between the stroma and the side of the tight junction towards the cleft of −1.5 mV (figure 5c). The resulting potential jump across the tight junction is 0.6 mV. The potential gradient along the cleft drives electro-osmotic flow via slip velocity. Indeed, as shown in figures 5d and 6a, the horizontal component of the velocity $u_{x}$ is not zero at y=±h/2, with fluid flowing along the lateral membrane, towards the tight junction.

**Table 4.** Fluxes computed using the permeabilities in set 1 (electronic supplementary material, table S4). Ion fluxes are in mol m^−2^ s^−1^. Lateral fluxes are $<J_{i}^{lc}>=\frac{2}{w}\int_{0}^{L}J_{i}^{lc}\text{d}x$, accounting for both half-clefts. The transendothelial fluxes are $J_{i}=J_{i}^{ca}+J_{i}^{TJ}$. Flow rates are in m^2^ s^−1^.

ion flux	Na^+^	K^+^	Cl^−^	HCO${,}_{3}^{-}$	water flow rate	
$J_{i}^{bc}$	$2.1\times 10^{-6}$	$2.6\times 10^{-6}$	$7.6\times 10^{-6}$	$6.5\times 10^{-6}$	$Q^{bc}$	$-4.5\times 10^{-14}$
$<J_{i}^{lc}>$	$9.4\times 10^{-7}$	$1.0\times 10^{-6}$	$3.6\times 10^{-6}$	$3.1\times 10^{-6}$	$Q^{lc}$	$8.9\times 10^{-14}$
$J_{i}^{ca}$	$3.1\times 10^{-6}$	$3.6\times 10^{-6}$	$1.1\times 10^{-5}$	$9.6\times 10^{-6}$	$Q^{ca}$	$4.5\times 10^{-14}$
$J_{i}^{TJ}$	$7.8\times 10^{-6}$	$-2.0\times 10^{-7}$	$-4.5\times 10^{-6}$	$-2.1\times 10^{-6}$	$Q^{la}$	$2.9\times 10^{-14}$
$J_{i}$	$1.1\times 10^{-5}$	$3.4\times 10^{-6}$	$6.8\times 10^{-6}$	$7.5\times 10^{-6}$	*Q*	$4.7\times 10^{-14}$

The directions in which ions (black arrows) and water (blue arrows) are moving across the cell membrane and the tight junction are shown in figure 6b, and the values of these fluxes are reported in table 4. The Na${,}^{+}$ flux across the tight junction is positive, thus Na${,}^{+}$ is going from the cleft to the anterior chamber, while all the other ions are crossing the tight junction in the opposite direction (negative fluxes). All the ion fluxes across the cell membrane are positive, thus ions enter the cell from the basolateral side of the membrane and they leave the cell across the anterior part of the membrane. The fact that ions move from the cleft to the cell is in agreement with the lower ion concentration in the cleft with respect to the stroma.

The qualitative behaviour of water fluxes across the cell membranes and the tight junctions is shown with blue arrows in figure 6b with the arrow lengths plotted at relative scales. Overall, integrating the fluid velocity from the cleft to the cell in [0,L], we get a positive flow rate: $Q^{lc}=2\int_{0}^{L}v_{osm}^{lc}\text{d}x=8.9\times 10^{-14}$ m${,}^{2}$ s^−1^ (table 4), so fluid enters the cell across the lateral membrane. The osmotic gradient between the cleft and the cell results in fluid flowing from the cleft into the cell in all the points where the cleft osmolarity is lower than the cellular one, which is almost everywhere. As a consequence, fluid flows from the stroma into the cleft, indeed the first point in figure 5d is $Q^{bl}>0$ (table 4). In the cleft, the longitudinal flux decreases (figure 5d), because part of the fluid is absorbed by the cell. Indeed, the velocity plot in figure 6a shows that the vertical component of the velocity $u_{y}$ is not zero at y=±h/2; therefore, some fluid is going from the cleft into the cell. There is also recirculation around x=L, with the longitudinal component of the velocity $u_{x}$ being negative in some points at the tight junction. However, there is a small net flow rate across the tight junction ($Q^{la}=2.9\times 10^{-15}$ m${,}^{2}$ s^−1^). Water flows into the cell from the cleft across the lateral sides of the membrane, and it leaves via the basal and apical membranes. Since we assume the solutions on the stromal side and on the apical side have the same composition, $Q^{ca}=-Q^{bc}$, and due to mass balance, $Q^{lc}=2Q^{ca}$. The overall transendothelial flow rate is $Q=Q^{ca}+Q^{la}=4.7\times 10^{-14}$ m${,}^{2}$ s^−1^, corresponding to a fluid flux of $\bar{Q}=\frac{Q}{w+h}=2.4\times 10^{-9}$ m s^−1^.

### 3.2. Sensitivity analysis

**Figure 7.**
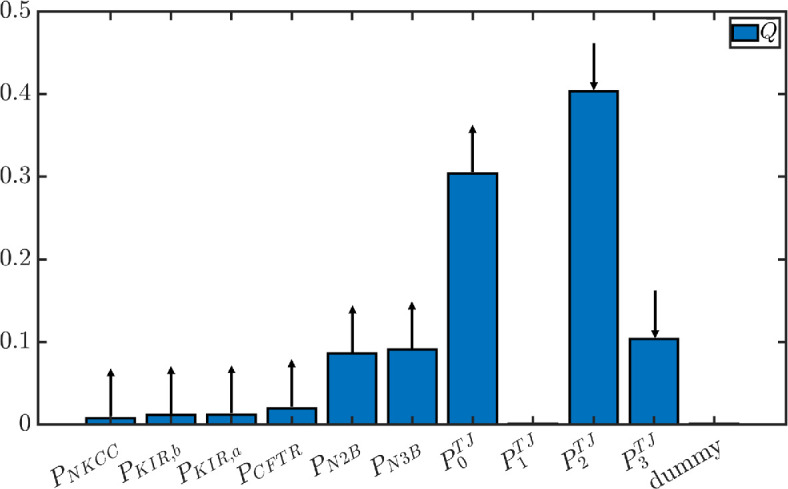
Total sensitivity indices for the parameters specified along the x-axis, where the considered output is the transendothelial water flux $\bar{Q}$. Upward-pointing (downward-pointing) arrows represent positive (negative) correlation between the parameter and the output variation.

From figure 7, the transendothelial water flux $\bar{Q}$ is mostly sensitive to the tight junction permeabilities of Na${,}^{+}$, Cl${,}^{-}$ and HCO${,}_{3}^{-}$. Conversely, it is not sensitive to the permeability to K${,}^{+}$ ($P_{1}^{TJ}$). Among the cellular transporters, N2B and N3B are those influencing fluid flux the most. There is a positive correlation between $P_{N2B}$, $P_{N3B}$, $P_{0}^{TJ}$ and $\bar{Q}$ (increasing those permeabilities results in an increased water flux), while the correlation between $P_{2}^{TJ}$, $P_{3}^{TJ}$ and $\bar{Q}$ is negative (increasing the permeability reduces the water flux). These correlations are indicated by the arrows on top of the bars in figure 7. In electronic supplementary material, section S9, we show more results from the sensitivity analysis: the results for the cellular concentrations are in electronic supplementary material, figure S6, those for the cellular and transendothelial potential in electronic supplementary material, figure S7, and those for the transendothelial ion fluxes in electronic supplementary material, figure S8.

## 4. Discussion

### 4.1. Summary

In this article, we present a mathematical model of fluid and ion transport across the corneal endothelium, accounting for two possible mechanisms explaining the endothelial pump: local osmosis and electro-osmosis. Our model accounts for four ion species (Na${,}^{+}$, K${,}^{+}$, Cl${,}^{-}$ and HCO${,}_{3}^{-}$) that move across the cellular membrane, through specific channels and transporters, while water movement is driven by osmotic and mechanical pressure differences. In the equations, we consider mass conservation for fluid and ions, conservation of momentum for fluid, electroneutrality and open circuit conditions. We derive two sets of equations: a system of ODEs describing the transport in the cleft between two adjacent cells and a system of algebraic equations for the transport in the cell, which we assume to be well mixed. Using an iterative procedure, we find a numerical solution satisfying both systems. The channel permeability parameters are obtained by least squares optimization, and their influence on ion concentrations, ion and water fluxes and electrical potential is assessed by a global sensitivity analysis.

### 4.2. Transendothelial water flux

**Figure 8.**
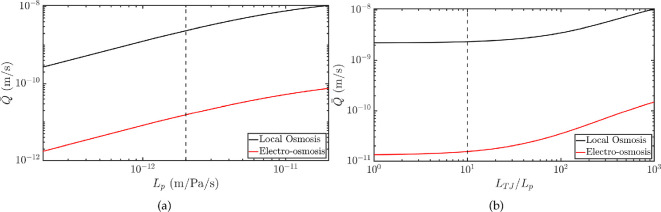
Comparison between the effect of electro-osmosis (red) and local osmosis (black) on the transendothelial water flux. The flux in the case of local osmosis was obtained by setting $C_{m}=\sigma_{0}=0$. The electro-osmosis effect was obtained by subtracting the local osmosis flux from the one with $C_{m}$ and $\sigma_{0}$ as in table 2 (which includes both effects). The permabilities are those in set 1. In (a), we plot the values of the water flux for different values of $L_{p}\in \left[2\times 10^{-13},2\times 10^{-11}\right]$ m Pa^−1^ s^−1^. The dashed line corresponds to the value $L_{p}=2\times 10^{-12}$ m Pa^−1^ s^−1^ used for the reference case. In (b), we plot the water flux against the ratio $L_{TJ}/L_{p}$. The value used in the reference case is $L_{TJ}/L_{p}=10$ (dashed line).

Our model predicts that both electro-osmosis and local osmosis generate water flux (volumetric flow rate per unit surface) in the right direction (from the stroma to the anterior chamber). With baseline values of the parameters, the magnitude of the flux is $2.4\times 10^{-9}$ m s^−1^, while in the literature it is reported to be approximately $10^{-8}$ m s^−1^ [46,47]. The magnitude of the transendothelial water flux is sensitive to the membrane permeability $L_{p}$ (figure 8a) and to the permeability of the tight junctions $L_{TJ}$, relative to the cell membrane (figure 8b). By increasing the reference value of $L_{p}$ by a factor of 10, the transendothelial water flux increases by a factor of 5. Instead, when we decrease the reference $L_{p}$ value by a factor of 10, the flux decreases by a factor of 9. In our reference case, we interpret the leakiness of the tight junction by assuming its permeability to be 10 times that of the cell membrane, but we did not find a precise value of $L_{TJ}$ in the literature.

In the model of [28] the authors found the water flux driven by local osmosis to be about $7\times 10^{-9}$ m s^−1^. The pump strength and membrane permeabilities to water in their model were comparable to the values used in this work. The authors pointed out that the tortuosity of the channel can increase its length by a factor of two. If we account for cleft tortuosity in our model and increase the cleft length from 5 to 12 μm as in [28], we find a water flux of $10^{-8}$ m s^−1^, which aligns well with the experimental observations. The authors also considered the hexagonal arrangements of the endothelial cells. In electronic supplementary material, section S2, we propose the computation of water flux accounting for hexagonal geometry and we show that for the reference parameter set this results in an increase of water flux by 8%.

From the sensitivity analysis, the parameters to which the transendothelial water flux $\bar{Q}$ is most sensitive are the tight junction permeabilities $P_{0}^{TJ}$, $P_{2}^{TJ}$ and $P_{3}^{TJ}$ to Na${,}^{+}$, Cl${,}^{-}$ and HCO${,}_{3}^{-}$. As shown in electronic supplementary material, figure S8, the correlation between the tight junction permeabilities and the water flux depends on the specific ion: it is positive for $P_{0}^{TJ}$, negative for $P_{2}^{TJ}$ and $P_{3}^{TJ}$. This can be explained by considering the effect of changing the membrane permeability on the cleft osmolarity. Indeed, since sodium flows out of the cleft across the tight junction (table 4), increasing its permeability decreases the cleft osmolarity. This, in turn, increases the osmotic jump between the cleft and the cell, resulting in a larger water flux. Conversely, chloride and bicarbonate flow into the cleft across the tight junction (table 4). Thus, increasing their permeability generates an increase in the cleft osmolarity and a corresponding decrease in the water flux.

We note that the model might overestimate the importance of the tight junction permeabilities. Indeed, the predicted current across the tight junction of 137 μA cm^−^${,}^{2}$ is higher than the experimentally estimated value 25.5 μA cm^−^${,}^{2}$ [29]. We selected the tight junction permeabilities using the fitting procedure described in electronic supplementary material, S4. The next iteration of the model that will include missing species and channels may result in a different optimal parameter set and resolve this inconsistency.

### 4.3. Role of electro-osmosis

The effect of electro-osmosis can be evaluated as the difference between the water flux computed with the parameters as in table 2 and that computed by imposing zero slip velocity ($C_{m}=\sigma_{0}=0$), therefore excluding electro-osmosis. Figure 8 shows that the effect of electro-osmosis on the transendothelial water flux is two orders of magnitude lower than the local osmosis effect, for all the values of hydraulic conductivity of the cell membrane, considered in the range $\left[2\times 10^{-13},2\times 10^{-11}\right]$ m s^−1^ (figure 8a), and for all the values of $L_{TJ}/L_{p}$, considered in the range [0.05,100] (figure 8b). The small effect of electro-osmosis compared to local osmosis, despite the slip velocity being non-negligible (figure 5d), is because the leading force driving fluid transport remains the osmotic pressure jump, which is almost unchanged by electro-osmosis. By averaging the results obtained for different values of $L_{p}$ as in figure 8a, the average difference between the osmotic and the mechanical pressure jump across the tight junction is $3.76\times 10^{3}$ Pa, with the average magnitude of the mechanical pressure being 5% the magnitude of the osmotic pressure. The effect of electro-osmosis increases the mechanical pressure, but on average this increase is 84 Pa, thus it is not sufficient to make it comparable to the osmotic pressure, which is almost unchanged.

**Figure 9.**
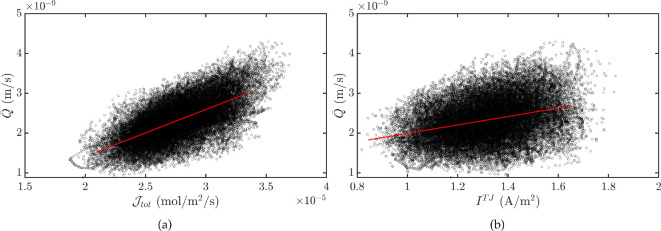
(a) Each point represents the value of the transendothelial water flux corresponding to a specific value of the transendothelial net ion flux $J_{tot}=\sum_{i=0}^{3}J_{i}$. The data were computed for the sensitivity analysis for all the parameters and chains. The red line is the linear fit of the data points. The slope is $1.2\times 10^{-4}$ m${,}^{3}$ mol^−1^. (b) Each point represents the value of the transendothelial water flux corresponding to a specific value of the paracellular current density $I^{TJ}=F\sum_{i=0}^{3}z_{i}J_{i}^{TJ}$. The data were computed for the sensitivity analysis for all the parameters and chains. The red line is the linear fit of the data points. The slope is $1.1\times 10^{-9}$ m${,}^{3}$ A^−1^ s^−1^.

Fischbarg & Diecke [29] modelled transport across the corneal endothelium by local osmosis and electro-osmosis. Water flux was assumed to be proportional to the net ion flux $J_{tot}$ so that $Q_{lo}=J_{tot}/C_{tot}$, where $C_{tot}$ is the total osmolarity in the extracellular space. Our model does not assume such a linear dependency, as we rigorously compute water flux, based on physical principles. In figure 9a, we show the scatter plot of water flux versus net ion flux predicted by our model. This confirms a positive relationship between these quantities even if we cannot claim a linear dependence since the cloud of points is sparse (coefficient of determination $r^{2}=0.4$). In particular, our sensitivity analysis shows that water flux is correlated with Na${,}^{+}$ and HCO${,}_{3}^{+}$ fluxes (electronic supplementary material, figure S9).

Similarly, in Fischbarg and Diecke’s model [29], the electro-osmotic water flux was taken to be proportional to paracellular current $I^{TJ}$ through an experimentally measured constant τ=2.37 μm cm${,}^{2}$ μA^−1^ h^−1^ $∼7.6\times 10^{-8}$ m${,}^{3}$ A^−1^ s^−1^ [16], so that $Q_{eo}=\tau I^{TJ}$. Evidence of this dependency is frequently used to infer that electro-osmosis is the main mechanism of fluid transport. This idea is possibly misleading, as we show in figure 9b that, even in the case of a negligible electro-osmotic effect (as predicted by our model), there is still a positive correlation between water flux and paracellular current. We note, however, that $r^{2}=0.1$ due to the high variance of the cloud of points, and that the constant of proportionality predicted by our model is significantly lower than the experimental value of τ [16]. This might be attributed to differences between the experimental setup (which also accounts for the stroma and an imposed current) and the assumptions underlying our model of the isolated endothelium.

**Figure 10.**
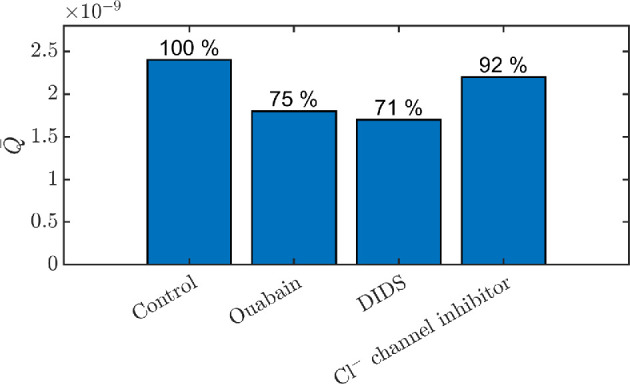
In this plot, we simulate the effects of inhibitors on the transendothelial water flux $\bar{Q}$ as in [29] by reducing by a factor of 2 the permeabilities of the channels affected by the inhibitors. Ouabain inhibits the Na${,}^{+}$–K${,}^{+}$ pump, DIDS inhibits N2B, N3B, AE,a, AE,b, CFTR and the Cl${,}^{-}$ channel inhibitor inhibits CFTR.

Fischbarg & Diecke [29] also reported the response of the water flux to different ion channel inhibitors, comparing it to experimental findings [43,65]. The effect of inhibition of specific channels in our model is shown in figure 10. Ouabain inhibits Na${,}^{+}$–K${,}^{+}$ ATPase and is reported to fully suppress water flux [65]. In our model, reducing pump permeability by a factor of 2 reduces water flux by 25%. DIDS inhibits all HCO${,}_{3}^{+}$ and Cl${,}^{-}$ transporters (except NKCC) [65], and we report that decreasing the amplitude of these channels by a factor of 2 leads to a reduction in water flux by 29%. The Cl${,}^{-}$ channel inhibitor was reported to reduce water flux by 14% [43], while in our model reducing CFTR permeability of a factor 2 results in an 8% water flux reduction. In [29], the authors could capture the experimentally measured reduction in water flux by DIDS and Cl${,}^{-}$ channel inhibitors only with electro-osmotic flux, while the inhibition by Ouabain was reproduced with both local and electro-osmotic models. Our model qualitatively captures the action of all these inhibitors on water flux, with local osmosis (standing gradient osmotic flow) being the leading flow mechanism.

### 4.4. Outlook

Although many of the model predictions are consistent with experimental measurements, some limitations could be addressed in future studies. First, the model does not include lactate, which is also present in high concentrations in the stroma (13 mM) and anterior chamber (7 mM) and may play a role in transendothelial water transport [6]. Second, the model does not include H${,}^{+}$ (protons) and does not address pH balance. Most experimental set-ups consider buffered solutions, and the present model performs well under these conditions since we prescribe concentration on both sides of the endothelium. However, when modelling physiological conditions, one has to account for pH balance, as net bicarbonate flux may result in pH increase. Furthermore, the model omits some relevant ion transporters, such as Na${,}^{+}$–H${,}^{+}$ exchanger (NHE) and possibly a H${,}^{+}$ pump included in [29]. Third, we do not consider the possibility of electro-osmosis across the tight junctions, which might play an important role [17,66].

This model can be considered as a first step towards a full model of water balance in the cornea, which, in addition to the endothelial pump should also account for the swelling pressure generated by the stroma. As a step forward, the inclusion of the metabolic species in our model could refine existing corneal swelling models [23–27], which do not capture the molecular mechanisms governing channels and transporters. A finer description of ion traffic across the endothelium, coupled with models of the other corneal layers, may provide insights into the effect of modifying transporter permeabilities on corneal hydration and swelling.

## Data Availability

The codes used for the results shown in the paper can be accessed at Zenodo [67]. Electronic supplementary material is available online [68].

## References

[1] Meek KM, Knupp C. 2015 Corneal structure and transparency. Prog. Retin. Eye Res. **49**, 1–16. (10.1016/j.preteyeres.2015.07.001) 26145225 PMC4655862

[2] Maurice DM. 1984 The cornea and sclera. In The eye (ed. H Davson), pp. 1–158, 3rd edn. Cambridge, MA: Academic Press. (10.1016/B978-0-12-206921-5.50006-4)

[3] Benedek GB. 1971 Theory of transparency of the eye. Appl. Opt. **10**, 459–473. (10.1364/AO.10.000459) 20094474

[4] Maurice DM. 1957 The structure and transparency of the cornea. J. Physiol. **136**, 263–286. (10.1113/jphysiol.1957.sp005758) 13429485 PMC1358888

[5] Narula P, Xu M, Kuang KY, Akiyama R, Fischbarg J. 1992 Fluid transport across cultured bovine corneal endothelial cell monolayers. Am. J. Physiol. **262**, C98–103. (10.1152/ajpcell.1992.262.1.C98) 1733238

[6] Li S, Kim E, Bonanno JA. 2016 Fluid transport by the cornea endothelium is dependent on buffering lactic acid efflux. Am. J. Physiol. Cell Physiol. **311**, C116–C126. (10.1152/ajpcell.00095.2016) 27225657 PMC4967133

[7] Bonanno JA. 2012 Molecular mechanisms underlying the corneal endothelial pump. Exp. Eye Res. **95**, 2–7. (10.1016/j.exer.2011.06.004) 21693119 PMC3199349

[8] Hedbys BO, Dohlman CH. 1963 A new method for the determination of the swelling pressure of the corneal stroma in vitro. Exp. Eye Res. **2**, 122–129. (10.1016/s0014-4835(63)80003-2) 13963667

[9] Midelfart A. 1987 Swelling pressure in bovine cornea determined by dialysis method. Acta Ophthalmol. **65**, 153–158. (10.1111/j.1755-3768.1987.tb06994.x) 3604605

[10] Hatami-Marbini H, Etebu E, Rahimi A. 2013 Swelling pressure and hydration behavior of porcine corneal stroma. Curr. Eye Res. **38**, 1124–1132. (10.3109/02713683.2013.809769) 23885800

[11] Olsen T, Sperling S. 1987 The swelling pressure of the human corneal stroma as determined by a new method. Exp. Eye Res. **44**, 481–490. (10.1016/s0014-4835(87)80159-8) 3595759

[12] Klyce SD, Dohlman CH, Tolpin DW. 1971 In vivo determination of corneal swelling pressure. Exp. Eye Res. **11**, 220–229. (10.1016/s0014-4835(71)80026-x) 5121743

[13] Ong Tone S, Kocaba V, Böhm M, Wylegala A, White TL, Jurkunas UV. 2021 Fuchs endothelial corneal dystrophy: the vicious cycle of Fuchs pathogenesis. Prog. Retin. Eye Res. **80**, 100863. (10.1016/j.preteyeres.2020.100863) 32438095 PMC7648733

[14] Szkodny D, Wróblewska-Czajka E, Wylęgała A, Wylęgała E. 2022 Indications and techniques of corneal transplants performed in one center in southern Poland, in the years 2001–2020. PLoS One **17**, e0276084. (10.1371/journal.pone.0276084) 36399464 PMC9674162

[15] Diamond JM, Bossert WH. 1967 Standing-gradient osmotic flow: a mechanism for coupling of water and solute transport in epithelia. J. Gen. Physiol. **50**, 2061–2083. (10.1085/jgp.50.8.2061) 6066064 PMC2225765

[16] Sánchez JM *et al*. 2002 Evidence for a central role for electro-osmosis in fluid transport by corneal endothelium. J. Membr. Biol. **187**, 37–50. (10.1007/s00232-001-0151-9) 12029376

[17] Rubashkin A, Iserovich P, Hernández JA, Fischbarg J. 2006 Epithelial fluid transport: protruding macromolecules and space charges can bring about electro-osmotic coupling at the tight junctions. J. Membr. Biol. **208**, 251–263. (10.1007/s00232-005-0831-y) 16648941

[18] Fischbarg J, Diecke FPJ, Iserovich P, Rubashkin A. 2006 The role of the tight junction in paracellular fluid transport across corneal endothelium. Electro-osmosis as a driving force. J. Membr. Biol. **210**, 117–130. (10.1007/s00232-005-0850-8) 16868674

[19] Lim JJ, Ussing HH. 1982 Analysis of presteady-state Na^+^ fluxes across the rabbit corneal endothelium. J. Membr. Biol. **65**, 197–204. (10.1007/BF01869963) 7062340

[20] Fischbarg J. 1972 Potential difference and fluid transport across rabbit corneal endothelium. Biochim. Biophys. Acta Biomembr. **288**, 362–366. (10.1016/0005-2736(72)90257-X) 5082997

[21] Barfort P, Maurice D. 1974 Electrical potential and fluid transport across the corneal endothelium. Exp. Eye Res. **19**, 11–19. (10.1016/0014-4835(74)90067-0) 4413199

[22] Hodson S. 1974 The regulation of corneal hydration by a salt pump requiring the presence of sodium and bicarbonate ions. J. Physiol. **236**, 271–302. (10.1113/jphysiol.1974.sp010435) 16992435 PMC1350802

[23] Klyce SD, Russell SR. 1979 Numerical solution of coupled transport equations applied to corneal hydration dynamics. J. Physiol. **292**, 107–134. (10.1113/jphysiol.1979.sp012841) 490333 PMC1280848

[24] Klyce SD. 1981 Stromal lactate accumulation can account for corneal oedema osmotically following epithelial hypoxia in the rabbit. J. Physiol. **321**, 49–64. (10.1113/jphysiol.1981.sp013971) 7338822 PMC1249613

[25] Li LY, Tighe BJ, Ruberti JW. 2004 Mathematical modelling of corneal swelling. Biomech. Model. Mechanobiol. **3**, 114–123. (10.1007/s10237-004-0054-7) 15378390

[26] Li LY, Tighe B. 2005 Numerical simulation of corneal transport processes. J. R. Soc. Interface **3**, 303–310. (10.1098/rsif.2005.0085) PMC1578740 16849239

[27] Leung BK, Bonanno JA, Radke CJ. 2011 Oxygen-deficient metabolism and corneal edema. Prog. Retin. Eye Res. **30**, 471–492. (10.1016/j.preteyeres.2011.07.001) 21820076 PMC4101817

[28] Liebovitch LS, Weinbaum S. 1981 A model of epithelial water transport. The corneal endothelium. Biophys. J. **35**, 315–338. (10.1016/s0006-3495(81)84792-3) 7272441 PMC1327525

[29] Fischbarg J, Diecke FPJ. 2005 A mathematical model of electrolyte and fluid transport across corneal endothelium. J. Membr. Biol. **203**, 41–56. (10.1007/s00232-004-0730-7) 15834688

[30] Dvoriashyna M, Foss AJE, Gaffney EA, Jensen OE, Repetto R. 2018 Osmotic and electroosmotic fluid transport across the retinal pigment epithelium: a mathematical model. J. Theor. Biol. **456**, 233–248. (10.1016/j.jtbi.2018.08.009) 30096403

[31] Bonanno JA. 2003 Identity and regulation of ion transport mechanisms in the corneal endothelium. Prog. Retin. Eye Res. **22**, 69–94. (10.1016/s1350-9462(02)00059-9) 12597924

[32] Kinsey VE. 1951 The chemical composition and the osmotic pressure of the aqueous humor and plasma of the rabbit. J. Gen. Physiol. **34**, 389–402. (10.1085/jgp.34.3.389) 14824506 PMC2147221

[33] Haber-Olguin A, Polania-Baron EJ, Trujillo-Trujillo F, Graue Hernandez EO. 2021 Thermographic behavior of the cornea during treatment with two excimer laser platforms. Transl. Vis. Sci. Technol. **10**, 27. (10.1167/tvst.10.9.27) PMC8399240 34427627

[34] Gavish N, Promislow K. 2016 Dependence of the dielectric constant of electrolyte solutions on ionic concentration: a microfield approach. Phys. Rev. E **94**, 012611. (10.1103/physreve.94.012611) 27575183

[35] Tuft SJ, Coster DJ. 1990 The corneal endothelium. Eye **4**, 389–424. (10.1038/eye.1990.53) 2209904

[36] Kreutziger GO. 1976 Lateral membrane morphology and gap junction structure in rabbit corneal endothelium. Exp. Eye Res. **23**, 285–293. (10.1016/0014-4835(76)90129-9) 976372

[37] Matthews GG. 2003 Cellular physiology of nerve and muscle. Oxford, UK: Blackwell. (10.1002/9781118687864)

[38] Rhee SW, Green K, Martinez M, Paton D. 1971 Water permeability of cat corneal endothelium in vitro. Invest. Ophthalmol. Vis. Sci. **10**, 288–293. 5549594

[39] Green K, Green MA. 1969 Permeability to water of rabbit corneal membranes. Am. J. Physiol. **217**, 635–641. (10.1152/ajplegacy.1969.217.3.635) 5807685

[40] Baum JP, Maurice DM, McCarey BE. 1984 The active and passive transport of water across the corneal endothelium. Exp. Eye Res. **39**, 335–342. (10.1016/0014-4835(84)90021-6) 6499954

[41] Mishima S, Hedbys BO. 1967 The permeability of the corneal epithelium and endothelium to water. Exp. Eye Res. **6**, 10–32. (10.1016/s0014-4835(67)80049-6) 6019476

[42] Sun XC, Bonanno JA, Jelamskii S, Xie Q. 2000 Expression and localization of Na^+^-HCO_3_^_-_^ cotransporter in bovine corneal endothelium. Am. J. Physiol. Cell Physiol. **279**, C1648–C1655. (10.1152/ajpcell.2000.279.5.C1648) 11029313

[43] Diecke FPJ, Wen Q, Sanchez JM, Kuang K, Fischbarg J. 2004 Immunocytochemical localization of Na^+^-HCO_3^-^_ cotransporters and carbonic anhydrase dependence of fluid transport in corneal endothelial cells. Am. J. Physiol. Cell Physiol. **286**, C1434–C1442. (10.1152/ajpcell.00539.2003) 14960417

[44] Bonanno JA, Srinivas SP, Brown M. 1995 Effect of acetazolamide on intracellular pH and bicarbonate transport in bovine corneal endothelium. Exp. Eye Res. **60**, 425–434. (10.1016/s0014-4835(05)80099-5) 7789422

[45] Rae JL, Watsky MA. 1996 Ionic channels in corneal endothelium. Am. J. Physiol. Cell Physiol. **270**, C975–C989. (10.1152/ajpcell.1996.270.4.c975) 8928754

[46] Kuang K *et al*. 2001 Corneal endothelial NKCC: molecular identification, location, and contribution to fluid transport. Am. J. Physiol. Cell Physiol. **280**, C491–C499. (10.1152/ajpcell.2001.280.3.c491) 11171568

[47] Diecke FP, Zhu Z, Kang F, Kuang K, Fischbarg J. 1998 Sodium, potassium, two chloride cotransport in corneal endothelium: characterization and possible role in volume regulation and fluid transport. Invest. Ophthalmol. Vis. Sci. **39**, 104–110. 9430551

[48] Jelamskii S, Sun XC, Herse P, Bonanno JA. 2000 Basolateral Na^+^-K^+^-2Cl^-^ cotransport in cultured and fresh bovine corneal endothelium. Invest. Ophthalmol. Vis. Sci. **41**, 488–495. (10.1152/AJPCELL.2000.279.5.C1648) 10670480

[49] Leuenberger PM, Novikoff AB. 1974 Localization of transport adenosine triphosphatase in rat cornea. J. Cell Biol. **60**, 721–731. (10.1083/jcb.60.3.721) 4274728 PMC2109228

[50] Tervo T, Palkama A. 1975 Electron microscopic localization of adenosine triphosphatase (NaK-ATPase) activity in the rat cornea. Exp. Eye Res. **21**, 269–279. (10.1016/0014-4835(75)90098-6) 126867

[51] McCartney MD, Wood TO, McLaughlin BJ. 1987 Immunohistochemical localization of ATPase in human dysfunctional corneal endothelium. Curr. Eye Res. **6**, 1479–1486. (10.3109/02713688709044512) 2827961

[52] Sun XC, Bonanno JA. 2002 Expression, localization, and functional evaluation of CFTR in bovine corneal endothelial cells. Am. J. Physiol. Cell Physiol. **282**, C673–C683. (10.1152/ajpcell.00384.2001) 11880256 PMC4100724

[53] Hanukoglu I, Hanukoglu A. 2016 Epithelial sodium channel (ENaC) family: phylogeny, structure–function, tissue distribution, and associated inherited diseases. Gene **579**, 95–132. (10.1016/j.gene.2015.12.061) 26772908 PMC4756657

[54] Ligocki AJ *et al*. 2021 Molecular characteristics and spatial distribution of adult human corneal cell subtypes. Sci. Rep. **11**, 16323. (10.1038/s41598-021-94933-8) 34381080 PMC8357950

[55] Lim JJ, Liebovitch LS, Fischbarg J. 1983 Ionic selectivity of the paracellular shunt path across rabbit corneal endothelium. J. Membr. Biol. **73**, 95–102. (10.1007/BF01870344) 6864769

[56] Probstein RF. 1994 Physicochemical hydrodynamics, an introduction. Hoboken, New Jersey: WileyBlackwell.

[57] Dvoriashyna M, Foss AJE, Gaffney EA, Repetto R. 2022 Mathematical models of water transport across ocular epithelial layers. In Modeling of mass transport processes in biological media (eds S Becker, AV Kuznetsov, F de Monte, G Pontrelli, D Zhao), pp. 405–433. Cambridge, MA: Academic Press. (10.1016/B978-0-323-85740-6.00002-9)

[58] Keener J, Sneyd J. 2009 Mathematical physiology I: cellular physiology. In Interdisciplinary applied mathematics (eds SS Antman, J Marsden, L Sirovich), pp. 367–390, vol. 2. New York, NY: Springer.

[59] Mori Y, Peskin C, Jerome J. 2007 A three-dimensional model of cellular electrical activity, pp. 367–390, vol. **2**. Taipei, Taiwan: Academia Sinica, Institute of Mathematics.

[60] The MathWorks. 2024 MATLAB version: 24.1.0.2568132 (r2014a). Natick, MA: The MathWorks Inc.

[61] Clenshaw CW, Curtis AR. 1960 A method for numerical integration on an automatic computer. Numer. Math. **2**, 197–205. (10.1007/BF01386223/METRICS)

[62] Sommariva A. 2013 Fast construction of Fejér and Clenshaw–Curtis rules for general weight functions. Comput. Math. Appl. **65**, 682–693. (10.1016/j.camwa.2012.12.004)

[63] Marino S, Hogue IB, Ray CJ, Kirschner DE. 2008 A methodology for performing global uncertainty and sensitivity analysis in systems biology. J. Theor. Biol. **254**, 178–196. (10.1016/j.jtbi.2008.04.011) 18572196 PMC2570191

[64] Saltelli A, Tarantola S, Chan KPS. 1999 A quantitative model-independent method for global sensitivity analysis of model output. Technometrics **41**, 39–56. (10.1080/00401706.1999.10485594)

[65] Kuang K, Li Y, Yiming M, Sánchez JM, Iserovich P, Cragoe EJ, Diecke FPJ, Fischbarg J. 2004 Intracellular [Na^+^], Na^+^ pathways, and fluid transport in cultured bovine corneal endothelial cells. Exp. Eye Res. **79**, 93–103. (10.1016/j.exer.2004.02.014) 15183104

[66] Fischbarg J, Hernandez JA, Rubashkin AA, Iserovich P, Cacace VI, Kusnier CF. 2017 Epithelial fluid transport is due to electro-osmosis (80%), plus osmosis (20%). J. Membr. Biol. **250**, 327–333. (10.1007/s00232-017-9966-x) 28623474 PMC5489618

[67] fede-vanone. 2025 fede-vanone/Interface_Corneal_endothelium_codes: codes for ‘A mathematical model of corneal endothelium pump function’ (v1.0). Zenodo. (10.5281/zenodo.15488402)40829637

[68] Vanone F, Foss AJE, Viola F, Repetto R, Dvoriashyna M. 2025 Supplementary material from: A mathematical model of corneal endothelium pump function. Figshare. (10.6084/m9.figshare.c.7863976)40829637

